# Rapid implementation of Veterans Health Administration telehealth creative arts therapies: survey evaluation of adoption and adaptation

**DOI:** 10.1186/s12913-023-09796-8

**Published:** 2023-07-19

**Authors:** Kristin M. Story, Mindy E. Flanagan, Sheri L. Robb, Dawn M. Bravata, Matthew J. Bair, David Otto, Teresa M. Damush

**Affiliations:** 1 Department of Veterans Affairs (VA) Health Services Research and Development (HSR&D) Center for Health Information and Communication, Richard L. Roudebush VA Medical Center, Indianapolis, IN 46202 USA; 2 Indiana University School of Nursing, Indianapolis, IN USA; 3 VA HSR&D Expanding Expertise Through E-Health Network Development (EXTEND) Quality Enhancement Research Initiative (QUERI), Indianapolis, IN USA; 4 Departments of Medicine and Neurology, Indiana University School of Medicine, Indianapolis, IN USA; 5 Regenstrief Institute, Indianapolis, USA; 6 Departments of Medicine, Indiana University School of Medicine, Indianapolis, IN USA; 7 VA Rehabilitation and Prosthetic Service, Recreation Therapy and Creative Arts Therapy Service, Washington, DC USA

**Keywords:** Telehealth, Creative arts therapy, Music therapy, Art therapy, Dance movement therapy, Drama therapy, Veterans, Observational study, Implementation

## Abstract

**Background:**

Creative arts therapies (CAT) are employed throughout the Veterans Health Administration (VHA) and are predominantly delivered in-person. Though telehealth delivery of CAT was used at several VHA facilities to increase services to rural Veterans, due to guidance from the Center for Disease Control and VHA that temporarily suspended or reduced in-person services, there was a large increase of CAT therapists enterprise-wide who adopted telehealth delivery. The aims of this study were to evaluate adoption and adaptation of CAT telehealth delivery and identify related barriers and facilitators.

**Methods:**

We deployed a survey guided by the Consolidated Framework for Implementation Research and administered it via email to all VHA CAT therapists (*N* = 120). Descriptive statistics were used to summarize data and responses were compared based on therapists’ age, years of experience and CAT discipline. Open survey field responses were summarized, qualitatively coded, and analyzed thematically.

**Results:**

Most therapists (76%) reported adopting telehealth with 74% each delivering > 50 CAT sessions in the prior year. Therapists adapted interventions or created new ones to be delivered through telehealth. Barriers included: technical challenges, control of the virtual space, and building rapport. Facilitators included added equipment, software, and infrastructure. CAT therapists adapted their session preparation, session content, outcome expectations, and equipment. CAT therapists reported being able to reach more patients and improved access to care with telehealth compared to in person visits. Additional benefits were patient therapeutic effects from attending sessions from home, therapist convenience, and clinician growth.

**Conclusions:**

VHA CAT therapists used their inherent creativity to problem solve difficulties and make adaptations for CAT telehealth adoption. Future studies may explore CAT telehealth sustainment and its effectiveness on clinical processes and outcomes.

**Supplementary Information:**

The online version contains supplementary material available at 10.1186/s12913-023-09796-8.

## Background

The Veterans Health Administration (VHA) is the largest integrated health care system in the United States serving more than six million enrolled Veterans each year [1]. The VHA provides a rich landscape to explore trends and lessons from health care delivery during the COVID-19 pandemic. Expansion of telehealth to improve access to care has been a long-standing VHA priority. With the onset of COVID-19, telehealth delivery became a necessity for many providers and patients. Rapid adoption of telehealth delivery was deployed as in-person outpatient visits at most VHA medical facilities were reduced or suspended for a specific period during lockdown restrictions [2, 3]. Telehealth visits in fiscal year (FY) 2021 increased 3,100% compared to FY2019 [1]. Many complimentary and integrative health therapies, including creative arts therapies, were deemed non-essential, were suspended from face-to-face visits, and directed to telehealth delivery. Due to rapid adoption, there was little time for preparation and telehealth training.

Creative arts therapies are complex interventions involving the use of art, dance and movement, drama, and music in a therapeutic relationship to address health and well-being. Creative arts therapies (CAT) pose unique challenges to telehealth delivery because interventions often involve more than verbal and visual interaction and require additional materials such as instruments, art supplies, and props used during a treatment session. For example, music therapy uses four primary types of methods: re-creative music experiences (singing songs), composition, improvisation, and receptive or listening experiences [4]. All of these are problematic or require some modification to be delivered virtually due to sound quality, lagging internet connection, and network latency. Other common barriers to telehealth delivery are internet access, lack of control over the environment, provider and patient lack of technical skills, and audio problems [5–7]. Technology skills in particular have been identified as a barrier to telehealth for older Veterans [7–9].

Literature about telehealth within CAT have identified benefits of increased access and continuity of services but also challenges such as limited technology skills among users, technology limitations, safety concerns (e.g., ensuring patients are in a safe and private environment), and therapeutic alliance [10–14]. Before the COVID-19 pandemic, telehealth CAT was rarely used across VHA and focused almost exclusively on reaching rural Veterans [15, 16]. According to administrative data provided by the VHA National Director of Recreation and Creative Arts Therapies, telehealth delivery of recreation therapies and creative arts therapies (CAT) increased by 1,271% in Fiscal Year (FY) 2020 (compared to 2019), and an additional 274% from FY2020 to FY2021. Simultaneously, in person delivery of CAT decreased 21% from FY2020 to FY2021. In fiscal year 2022, telehealth CAT delivery is on track to match or exceed FY2021.

Although CAT telehealth has increased, there is a lack of health system-wide evaluations of therapists’ telehealth implementation. We believed this evaluation would help us to better understand delivery differences between in-person and telehealth CAT and factors that may influence successful implementation to ultimately improve CAT telehealth delivery and patient access. To guide our evaluation, we utilized The Consolidated Framework for Implementation Research (CFIR). The CFIR provided a systematic approach to assess implementation context as well as potential barriers and facilitators of effective implementation [17].

The objective of this study was to conduct a formative developmental evaluation of the VHA’s CAT telehealth delivery to Veteran patients. We used the CFIR as a framework to guide our interview guide and analysis [17, 18]. The primary aims were twofold: to describe the adoption and adaptations of CAT telehealth delivery to Veteran patients during the COVID-19 pandemic; and to identify barriers and facilitators to delivering telehealth CAT to Veteran patients to inform future delivery and improve patient access.

## Methods

We obtained Institutional Review Board approval from Indiana University IRB# 2,011,894,227 and the VHA Research and Development committee to conduct this research. Signed informed consent was obtained by each participant.

We employed a survey with quantitative and qualitative responses to evaluate CAT practices in telehealth service delivery to Veteran patients. We collected data on therapists’ experience though a survey deployed in May 2021 nation-wide to all VHA Creative Arts Therapists (*n* = 120). Two weekly reminders were sent after the initial invitation and individualized messages were sent to non-responders. The survey included closed and open-ended questions and was guided by the following CFIR domains (constructs): intervention characteristics (adaptability of CAT experiences), outer setting (Veteran needs and resources), inner setting (structural characteristics and readiness for implementation), and characteristics of therapists (knowledge and beliefs about telehealth CAT, self-efficacy) [17]. The survey consisted of 25–34 questions and was administered using VA REDCap. Branching logic was used to tailor survey questions according to therapist responses. For example, if a therapist answered that they had never delivered a telehealth CAT session, they skipped questions pertaining to virtual delivery experiences (See Supplemental Material for Survey). Prior to administering the survey to VA CAT therapists, we pre-tested the questionnaire with five non-VA CAT therapists who had been using telehealth delivery. We administered the questionnaire through individual interviews to obtain immediate feedback about the proposed questions. Pre-testing feedback revealed that the questions were clearly articulated and relevant to CAT therapists. However, based on the detail in the answers provided, we expanded the number of questions on technology, types of interventions, and adaptations.

### Data analyses

Descriptive statistics (frequencies and percentages) were calculated for all questions with quantitative response items. Based on our research protocol, one intent was to explore respondent characteristics. Comparisons were conducted between CAT therapists’ characteristics (i.e., age, years of experience) and their training needs and confidence using technology; and between the CAT type (music or art) and technology issues specific to discipline. Chi-square tests, Fisher’s exact tests, and generalized linear models were used to test for differences as appropriate for variable types (categorical or continuous) and cell size. Analyses were conducted using SAS software version 9.4 (Cary, NC). Copyright © 2013 SAS Institute Inc.

**Fig. 1:**
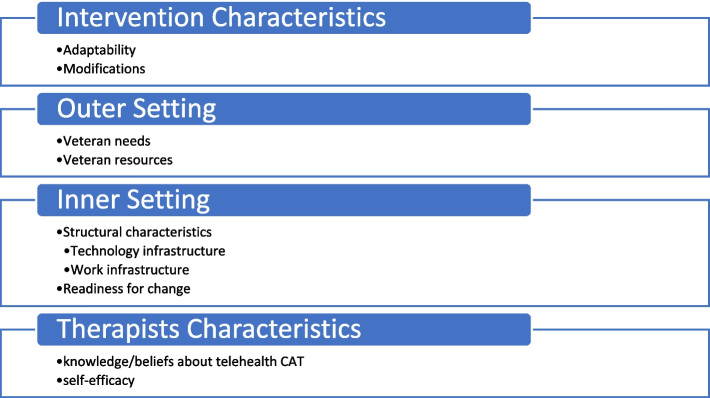
CFIR domains and constructs that guided interview and analysis

Qualitative analyses began with a deductive approach to thematic analysis for the open-ended responses, using Braun and Clarke’s method of thematic analysis.[19] Deductive codes were taken directly from the interview questions, which were guided by the CFIR constructs and domains (see Fig. 1). Responses were exported into Microsoft Excel, extracting key phrases related to the research questions, and allowing for identification of new codes not included in the original deductive codes. Two investigators independently coded the open-ended responses. From the coded data, themes were iteratively developed. Themes were discussed by a minimum of two research team members and further reviewed by the entire team to obtain consensus.

## Results

### Sample characteristics

The survey response rate was 77% (92/120). Personalized reminders increased the initial response rate from 60 to 77%. Respondents were comprised of board-certified music therapists (61%), board certified or registered art therapists (35%), board certified or registered dance movement therapists (4%) and registered drama therapists (1%), which mirrors the composition of CAT therapists employed by the VHA. Survey responders represented 17 of the 18 VHA regions of the United States. Sample characteristics are outlined in Table 1.

**Table 1:** Sample characteristics

Characteristic	Survey Frequency n (%) (*n* = 92)
Gender	
Female	70 (76.1)
Male	16 (17.4)
Missing	6 (6.5)
Race	
White	81 (88.0)
American Indian or Alaska Native	2 (2.2)
Asian	1 (1.1)
Missing	8 (8.7)
Ethnicity	
Not Hispanic or Latino	76 (82.6)
Hispanic or Latino	7 (7.6)
Missing	9 (9.8)
Education	
Bachelor's Degree	27 (29.4)
Master's Degree	59 (64.1)
Doctorate level degree	6 (6.5)
Specialty	
Music Therapy	56 (60.87)
Art Therapy	32 (34.78)
Dance/Movement Therapy	4 (4.35)
Drama Therapy	1 (1.09)
Years delivering CAT	
More than 15 years	34 (37.0)
11–15 years	28 (30.4)
6–10 years	18 (19.6)
Less than 5 years	12 (13.0)

### Telehealth adoption and characteristics for creative arts therapies

As shown in Table 2, 69 (75.8%) therapists delivered a telehealth CAT session between May 2020-May 2021, and about 74% of those each delivered more than 50 sessions that year. Most therapists (59/69, 85.5%) used VA Video Connect (VVC), the internal VHA platform for delivering telehealth services from the clinician at one location directly to the patient’s home. However, for part of the pandemic other video platforms (e.g., WebEx, Zoom) were temporarily approved and used by therapists. Therapists were asked to provide further explanation for any preferred platforms. Many comments were positive or neutral; however, of the 17 negative comments, 12 (70.5%) were about VVC, primarily related to poor audio quality and limited capability for group therapy sessions. The Zoom platform received the most positive comments citing it produced a better music experience and could display all Veterans participating in a group.

**Table 2:** Telehealth usage characteristics

Characteristic	Frequency (%)
**Questions only for CAT therapists who used telehealth (*n* = 69)**	
Number of telehealth CAT sessions provided in the previous year	
More than 50	51 (73.9)
Between 21–50	8 (11.6)
Between 11–20	2 (2.9)
Less than 10	8 (11.6)
Platforms used for telehealth CAT	
VVC	59 (85.5)
Telephone	33 (47.8)
Zoom	32 (46.4)
WebEx	26 (37.7)
Technical ease/ difficulty with telehealth delivery	
Very few or no issues	16 (23.2)
Moderate level of issues	45 (65.2)
High level of issues	8 (11.6)
Adaptations and modifications for telehealth delivery	
Modified techniques to deliver interventions/directives	32 (46.4)
Created new interventions/directives	27 (39.1)
Delivered same interventions/directives as in person	10 (14.5)
Outcomes/goals changed for telehealth sessions	
No	46 (66.7)
Yes	23 (33.3)
**Questions for all CAT therapists (*n* = 91)**^**a**^	
Have you delivered a telehealth CAT session in the previous year?	
Yes	69 (75.8)
No	22 (24.2)
Types of training requested	
Basic training	22 (24.2)
Hands-on training	40 (44.0)
Examples from CAT therapists	77 (84.6)
Proper infrastructure to successfully deliver telehealth	
Not at all	10 (11.0)
Some, but could be improved	59 (64.8)
Yes	17 (18.7)
Unsure	5 (5.5)
Administrative support to successfully deliver telehealth	
No	16 (17.6)
Some, but could use more	38 (41.8)
Yes	28 (30.8)
Unsure	9 (9.9)

^a^One participant did not answer

#### Technology problems and challenges

Most therapists (65%) reported moderate technology issues that they were able to overcome. Technology issues were often specific to the creative arts discipline with 76.3% (29/38) of music therapists reporting technology problems related to their specific therapy modality compared to 29.6% of art therapists (8/27) (*p* < 0.001).

Technology issues were attributed most often to platform or internet issues (57%) and difficulties from Veterans limited technology skills (45%). As shown in Table 3, therapists’ age was related to training needs (*p* < 0.002) and confidence using technology (*p* < 0.002). Therapists aged 55 years and over reported the highest need for training and lowest average technology confidence. Therapists with 6–10 years of experience reported the lowest rate of training needs and highest technology confidence, but only technology confidence reached statistical significance.

**Table 3:** Therapist characteristics and virtual CAT training needs and technology confidence

Variable	Training needs (Yes)		Confidence using technology	
	Count (%)	*p*-value	Mean (SD)	*p*-value
Age (years):				
25–34	5/25 (20.0)	.002	77.44 (17.9)	.002
35–44	10/25 (40.0)		73.20 (19.2)	
45–54	2/13 (15.4)		77.08 (21.2)	
55 +	14/20 (70.0)		54.60 (25.7)	
Experience (years):				
< 5	5/12 (41.7)	.06	74.83 (22.2)	.049
6–10	2/18 (11.1)		80.56 (15.5)	
11–15	12/28 (42.9)		72.61 (18.9)	
> 15	15/33 (45.5)		63.52 (26.4)	

**Fig. 2:**
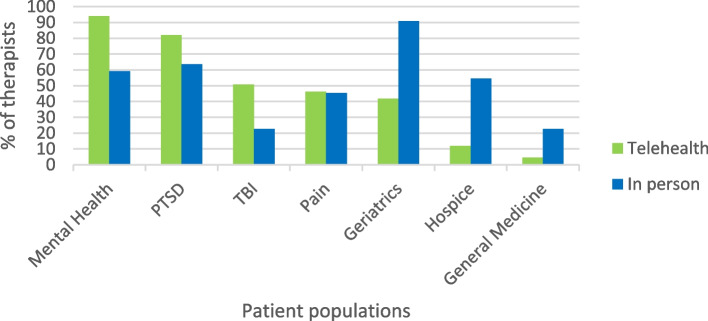
Patient populations receiving telehealth and in-person CAT

Therapists delivered telehealth CAT in outpatient and inpatient settings to a variety of populations, with 81% working with multiple populations. Telehealth CAT was prominently delivered to Veterans with mental health issues (93%) and post-traumatic stress disorder (PTSD) (81%). Therapists also delivered telehealth CAT to Veterans with traumatic brain injury (TBI), pain, geriatric populations, hospice, and surgery or general medicine support. Therapists who had not delivered telehealth sessions differed in their patient populations treated, with 90.9% working with older adult patients (see Fig. 2). In response to whether there is a need for telehealth CAT delivery, 98% of the therapists said yes, although some listed certain caveats such as *only when in-person delivery is not possible* (9/91, 9.9%), or *only for certain patient populations* (12/91, 13.2%).

#### Thematic results of survey open responses

Thematic analysis from survey open responses revealed therapists’ perceived barriers, adaptations needed for effective telehealth, and benefits to telehealth delivery. Survey themes and exemplar quotes are presented in Table 4. The results narrative below provides more detail regarding the results.

**Table 4:** Survey themes and quotes

Category	Theme	Example
Barriers	Theme 1: Barriers to telehealth CAT delivery are limitations of equipment and platforms, and lack of Veteran resources and technical skill	“*Syncing of sound is the major one, but also lack of instruments for patients and their internet connection being slow.”* [survey 84, music therapist]
	Theme 2: Less control over the virtual space including therapist reliance on Veteran to find a private, quiet space that is safe and contains the needed materials for a CAT session	*“not really knowing what is going on outside of the camera despite asking them to have a place where they feel comfortable (privacy).”* [survey 88, art therapist]
	Theme 3: Building rapport is more difficult through telehealth CAT due to interaction that is limited, less nuanced, and personal	*“It feels less personal; not as easy to build therapeutic rapport.”* [survey 62, music therapist]
Adaptations to CAT sessions	Theme 1: Therapists are adapting how they prepare for sessions by using materials from Veterans’ home, mailing materials in advance, and relying on more verbal instructions compared to in person sessions	*“I'm having to consider how to best offer services using the client's available resources.”* [survey 10, music therapist]
	Theme 2: Therapists are modifying the CAT experiences to include less synchronous music making, restricted range of movement to ensure safety, and more reliance on Veterans learning the art and music experiences independently of hands-on instruction	*“Adapted live engagements to account for latency issues and given veteran more opportunities to be a leader/communicate in a different way during sessions.”* [survey 9, music therapist]
	Theme 3: Due to Veterans’ needs during the COVID-19 pandemic, therapists modified goals to a general focus on self-care and maintaining social interactions	*“I am finding that the goal of staying connected with others and not isolating has risen to the top of the priority list for many of my patients.”* [survey 69, music therapist]
Adaptations to facilitate telehealth delivery	Theme 1: The addition of audio interfaces, cameras and lighting improves the quality and ease of delivery of telehealth CAT	*“using an audio interface to improve instrument playing audio.”* [survey 28, music therapist]
	Theme 2: Additional tools through software and screen sharing enhances visuals and music sharing to facilitate easier delivery and improve quality of telehealth CAT interactions	*“Screen sharing has been a support to art therapy services as reference images, how-to videos, websites, tutorials and other resources can be easily shared in real time.”* [survey 19, art therapist]
	Theme 3: Providing telehealth CAT from the provider’s home increases the number of patients to receive services	*“I do not have assigned space and have bounced from conference room to conference room to have programming. With virtual care I can see twice as many groups without space limitations.”* [survey 4, music therapist]
Benefits	Theme 1: Telehealth Improves access and reach by providing continuity of care, and reaching Veterans who are homebound, or live far from nearest medical facility	*[Benefit of telehealth is..] “being able to see a larger number of veterans in the community that might not be seen otherwise due to transportation issues or scheduling.”* [survey 76, music therapist]
	Theme 2: Telehealth provides additional therapeutic benefits from Veterans being able to attend sessions from home while maintaining social connections	*“Patients are able to engage from their own home and are creating healing environments within their spaces.”* [survey 23, art therapist]
	Theme 3: Telehealth delivery provides enhanced therapist creativity, improves scheduling flexibility and makes more efficient use of therapists’ time	*“I have felt challenged to move out of my comfort zone and I feel that's made me grow as a clinician.”* [survey 10, music therapist]

#### Barriers to telehealth delivery

Barriers to telehealth delivery fell under three main themes: technical, space, and rapport. Technical barriers were mentioned by 70% (46/66) of therapists. Video platform limitations were reported to cause a significant decrease in sound quality for synchronous music making. Some Veterans did not have internet access in their home or had outdated digital devices. Equipment that could improve session quality (e.g., better lighting, additional cameras, microphones, and external speakers) was sparse for therapists and not available to most Veterans. Poor internet connectivity or inadequate bandwidth was an issue for therapists and Veterans. Knowledge and skills to navigate changing technology was a barrier for many Veterans, and therapists needed to dedicate session time to teach technology when issues arose.

Another issue mentioned by therapists was that there was less control over safety and privacy due to lack of control over the Veteran’s environment. Dance and movement experiences were limited in range or therapists needed to spend extra time assessing the Veteran’s home environment for adequate space. Not all Veterans had a private space where they could participate in sessions.

Therapists also detailed the difficulty of building rapport through telehealth sessions. The absence of physical presence and the limited ability to see and hear parts of treatment sessions contributed to lack of connection, ability to offer support, and limited information used to gain insight into the therapeutic process.

#### Therapist adaptations to CAT

Most therapists (85.5%) who delivered telehealth CAT, adapted or created new interventions for delivery. For example, therapists relied more on verbal communication, needed to plan more, and shifted from shared synchronous experiences to asynchronous experiences. Adaptations and modifications occurred in how they shared materials with Veterans, the individual CAT experiences, and overall expectations regarding process and outcomes.

Session planning for telehealth CAT occurred earlier than for in-person sessions so that art supplies, instruments, or drama props could be mailed in advance. Therapists who could not mail supplies relied on resources in the Veterans’ environment, such as making instruments or props from household items. Without the ability to provide hands-on modeling, there was more reliance on verbal instructions.

Art therapists noted the limited camera view during telehealth sessions and how that altered art-making, increased verbal communication, and necessitated more independent work time for the Veteran. Virtual platforms were not designed for shared music making, thus the quality and opportunity for music interactions was negatively affected. Therapists needed to adapt experiences so only one person was heard at a time or group playing occurred with microphones muted, meaning individuals could see that they were playing with the group but could only hear themselves. Music therapists added more receptive interventions, which had fewer challenges than active ones but still needed modification, for example providing guidance before or after rather than during the music.

Therapists altered their expectations of session content and depth of work. Goals were modified to be more supportive and focused on self-care to meet Veterans’ needs during the pandemic. Treatment goals also shifted due to concerns about safety when engaging in issue-oriented work. Therapists felt that certain advanced CAT methods (e.g., Guided Imagery and Music, Authentic Movement) required greater presence and therapeutic support not possible over telehealth. One therapist shared, *“There is less opportunity for experiential interventions that promote in-session catharsis, for example, I am unable to provide the option for Veterans to "make a mess" in a safe space and have the therapeutic outcome of letting go, destroying something or engaging with messiness/chaos, and working through the aftermath. This has considerably adjusted the scope of my art therapy interventions.”* [survey 6, art therapist].

When possible, therapists adapted equipment on their end to improve telehealth delivery. Software resources that enhanced visuals or enabled song sharing improved the quality of the online experience and provided tools that were not possible in person. Additional equipment and software were often dependent on resources provided by clinic/hospital administration. Therapists stated that CAT delivery was easier when internet bandwidth was adequate, and space was provided for conducting telehealth sessions. VHA facilities have limited office space. For some therapists, the option to work remotely meant they had a dedicated quiet home office where they could deliver sessions to more Veteran patients.

#### Benefits

More than two-thirds (68.7%) of therapists stated that they were able to provide services to more Veterans. Access improved because transportation barriers (lack of car, parking difficulties) were removed. Veterans could access services despite debilitating conditions that prevented them from leaving home, and they could continue care throughout the COVID-19 pandemic. Reach was improved when, through telehealth, CAT could be expanded to areas where facilities did not have CAT therapists, or into rural areas removed from VA Medical Centers. Veterans who preferred telehealth CAT cited scheduling convenience, especially for younger Veterans working or in school.

Veterans reported to therapists that they appreciated staying connected during the pandemic and attending sessions from home, especially those who had social anxiety. These were issues of convenience and therapeutic benefits. Veterans attending sessions from home provided a context of social and family environment that helped build rapport during sessions.

Therapists reported their time was used more efficiently because they did not need to set up equipment or session rooms. Some therapists gained time previously spent traveling between VHA facilities, which added flexibility with scheduling. Being pushed outside their comfort zone was cited as an opportunity for clinician growth by several therapists. The addition of screen sharing and software led to exploration of new content such as virtual museum visits and symphony concerts.

## Discussion

Similar to recent findings on mental health providers and telehealth delivery, there were differences across therapist characteristics and telehealth adoption [20, 21]. Age and years of experience were related to training needs and confidence using technology. Our thematic results for telehealth facilitators and barriers are similar to those identified in other studies that examined music therapy and other specialty fields, including: the ability to expand reach, technology barriers, and therapeutic rapport. Similar findings from a systematic review in the mental health field were that remote care made therapy more accessible, and barriers were related to service users resources and technology skills, and the ability to develop rapport in remote sessions [22]. A scoping review of how music therapists adapted to providing remote sessions mirrored some of our adaptation themes: changes in therapeutic goals, preparation of clients for sessions, and the importance of flexibility [23]. Our findings also provide a unique perspective of challenges and adaptations required for working with Veterans and other specialty areas that may rely on non-verbal interactions.

### Complexity of telehealth delivery

Identified facilitators for some therapists emerged as challenges for others. For example, some therapists appreciated the efficiency of telehealth (e.g., removed commute time between sites, or the need to set up rooms), while others found the added time for technical issues or mailing art materials to be less efficient. Some appreciated having a view into Veterans’ homes and resources, but others found the lack of control over the environment challenging. With the absence of hands-on assistance, therapists reverted to verbal cuing or instructions. The reliance on verbal communication conflicts with one of the tenants of CAT, an emphasis on creative process rather than verbal interactions. Self-expression through music, art, movement, and drama rely on play and being in the creative process together. Creative arts therapies involve a triangular relationship among the therapist, the patient, and the creative art process. The transfer of this relationship to a virtual environment is an added complexity due to the sensorial component of engaging in the arts and how that may differ in-person and online. Despite these process challenges, therapists appreciated the session continuity and the ability to provide social connection during isolation. Therapists recognized that telehealth may not be appropriate for everyone but improves access for many Veterans.

### Limitations

Results from this study are limited to the experiences of VHA CAT therapists and may not reflect practices in other settings. Most survey responses were from CAT therapists who had used telehealth (75.8%) and therefore we may have failed to capture the concerns of therapists who have not adopted telehealth. Unique to this study is the perspective of therapists across CAT disciplines who work with multiple Veteran patient populations across a National Healthcare System. Of note, the VHA has relatively few dance/movement (4%) and drama therapists (1%), so the views represented here are skewed towards art and music therapy. The comparison of characteristics to various results was a post-hoc analysis and is subject to bias. Nonetheless this study evaluated the shift from in person to virtual CAT in the VA across a national VHA healthcare system and we compared CAT characteristics associated with that shift through post-hoc analyses. We learned about adaptations but not how that affected clinical processes and outcomes, which was outside the scope of this study. We did not collect patient-reported outcomes or assess the perceptions of Veterans, which are directions for our future research.

### Contributions to the literature

With the onset of COVID-19, telehealth was adopted across many health services, but some fields lacked evidence-based guidelines to inform telehealth delivery. Evaluating the process of rapid implementation provides recommendations for moving forward. We found that Art, Dance/movement, Drama, and Music Therapists encounter unique challenges that require adaptations for telehealth delivery because of sound and visual issues when using music, art, and movement interventions. This research contributes to recognized literature gaps, including barriers and facilitators to implementation of telehealth creative arts therapies, and adaptations to improve quality of delivery. Findings may be useful to other Complementary and Integrative services.

### Best practices

Based on therapists’ feedback, there are several recommendations for improved telehealth CAT delivery. The recommendations are not discrete themes but often overlap with themes. To highlight that connection, the category and themes are identified in parenthesis following each recommendation.

#### For therapists:

- Allow more time for preparation and planning to test technology, equipment, and provide session materials to Veterans through mail or email (barriers, theme 1; adaptions to sessions, theme 1).
- Use creativity and available software to enhance visuals and music sharing capabilities (adaptations to facilitate delivery theme 2; benefits, theme 3).
- Provide guidelines to Veteran patients, to help them understand how they can prepare their environment to engage in telehealth CAT. Examples include having a private space, having provided materials nearby, and removing obstacles for dance/movement experiences (barriers, theme 2; adaptations to sessions, theme 2).
- Maintain flexibility regarding expectations and goals when sessions are delivered via telehealth. For example, time may need to be spent on technology issues that emerge (barriers, theme 1; adaptations to sessions, theme 3).
- Connect Veteran patients with needed resources to engage in telehealth such as VHA consults to provide digital tablets or internet-enabled tablets, and technology training (barriers, theme 1).

#### For administration:

- Ensure that therapists have the proper equipment and office space to deliver telehealth sessions (barriers, theme 1).
- If the facility does not have adequate space and resources, consider remote work options (adaptations to facilitate delivery, theme 3; benefits, theme 3).
- Provide training and support for therapists. Specifically, CAT therapists are asking for training and examples from other CAT therapists (adaptation to facilitate delivery, theme 1).
- Provide resources (equipment, training, and support) for Veterans (barriers, theme 1; benefits, theme 1 & 2.

## Conclusion

One year into the COVID-19 pandemic, CAT therapists reported on their experiences with rapid telehealth adoption. Our results demonstrated that the majority had adopted telehealth delivery. There were significant differences in training needs and confidence levels among different age groups and music therapists encountered a significantly higher amount of technology issues compared to art therapists. Thematic analysis from open-field responses revealed facilitators, benefits, barriers, and adaptations for telehealth delivery.

Because of process limitations related to technology, telehealth CAT experiences were often adapted from synchronous to asynchronous, potentially changing the therapeutic process and outcomes. Future research should include clinical trials to assess differences in outcomes when CAT is delivered via telehealth compared to in person delivery.

## Supplementary Information

**Additional file 1: Supplementary file.** Telehealth creative arts therapy survey.

## Data Availability

The dataset is not publicly available due to confidentiality policies.

## Abbreviations

CAT: Creative Arts Therapies
PTSD: Post traumatic stress disorder
TBI: Traumatic brain injury
VHA: Veterans Health Administration
VVC: VA Video Connect
