# Supplementary material for: Rapid implementation of Veterans Health Administration telehealth creative arts therapies: survey evaluation of adoption and adaptation
Source: BMC Health Serv Res. 2023 Jul 19;23:769. doi: 10.1186/s12913-023-09796-8 (PMC10357876; doi:10.1186/s12913-023-09796-8)
Supplement: Supplementary file 1 — Additional file 1: Supplementary file. Telehealth creative arts therapy survey. [file 12913_2023_9796_MOESM1_ESM.docx]

Supplementary file. Telehealth Creative Arts Therapy Survey

Implementation of Virtual CAT (Creative Arts Therapy)

**Demographic Questions**

1. What is your area of specialty in CAT (choose all that apply)?
   1. Art Therapist
   2. Dance/ Movement Therapist
   3. Drama Therapist
   4. Expressive Arts Therapist
   5. Music Therapist
   6. Other (specify) _____________________
2. Please select your highest degree/ level of education.
   1. Bachelor’s Degree
   2. Master’s Degree
   3. Doctorate level degree (PhD, EdD, DBA, etc…)
3. Have you completed any advanced trainings or fellowships? If so, what and when?
4. Please select your current work setting (choose all that apply).
   1. Inpatient hospital
   2. Inpatient mental health
   3. Outpatient
   4. Multiple settings through referrals
   5. Other (specify) _______________
5. Please select type of sessions you provide (choose all that apply).
   1. Individual CAT sessions
   2. Group CAT sessions
   3. In-person CAT sessions
   4. Virtual CAT sessions
6. How many years have you been a practicing CAT?
   1. Less than 5 years
   2. 6-10 years
   3. 11-15 years
   4. More than 15 years
7. Please select your gender.
   1. Female
   2. Male
   3. Other (specify) __________________
8. What is your age today?
9. Race, (standardized categories)
   1. American Indian or Alaska Native
   2. Asian
   3. Black or African American
   4. Native Hawaiian or Other Pacific Islander
   5. White
10. Ethnicity
    1. Hispanic or Latino
    2. Not Hispanic or Latino

**Topic: Use of Telehealth - In the next set of questions, we are asking about your experience specifically** **since March 2020.**

1. Have you delivered a CAT session virtually since March 2020?
   1. Yes (continue with next question)
   2. No (skip to question 14)
2. Please choose approximately how many virtual CAT sessions you have delivered since March 2020.
   1. Less than 10
   2. 11-20
   3. 21-50
   4. More than 50
3. What platforms have you used for virtual CAT sessions? (choose all that apply)
   1. VVC
   2. Doxy Me
   3. Doximity
   4. Skype
   5. Zoom
   6. Telephone
   7. Other (specify) ___________________

If you prefer certain platforms, please list here: ___________

1. Please choose your overall level of technical ease/ difficulty with virtual delivery.
   1. I have experienced very few or no issues with technology.
   2. Moderate level of problems with technology, but able to overcome.
   3. High level of problems with technology that led to termination of chosen intervention or session.
2. The technology issues were related to (choose all that apply):
   1. patient perspective/ use
   2. therapist perspective/ use
   3. Both
   4. Neither (e.g. related to platform or internet issues outside of user’s control)
3. Were any of the technology issues specific to your CAT discipline (e.g. process of music making, art making, etc.)
   1. Yes
   2. No
4. What has been the most difficult aspect of Virtual CAT delivery?
5. What has been the best aspect of Virtual CAT delivery?
6. What clinical patient populations have you worked with, using virtual delivery since March 2020? (choose all that apply)
   1. Mental health
   2. PTSD
   3. TBI
   4. Pain
   5. Hospice
   6. General Medical/ procedural support
   7. Geriatrics
   8. Other (specify) _________________
7. Are most of the interventions for which you deliver virtually?
   1. Active interventions (where the participant is actively creating, such as drawing, making music, dancing)
   2. Receptive interventions (e.g. listening to music, viewing images)
   3. About equal active and receptive
8. To what extent have you modified or adapted interventions for virtual delivery?
   1. Not at all, I am able to do the same interventions I did in person
   2. I have adapted new techniques that allow me to deliver the same in person interventions virtually
   3. I have developed new interventions for virtual delivery

If answer to question 11 was “b”, how did you adapt or modify?

1. Have you needed to change any outcomes or goals that you’ve found are better or more challenging to address in virtual sessions?
   1. Yes
   2. No

If answer to 12 was yes, please provide an example.

1. Have you ordered a digital divide consult for a Veteran?
   1. Yes
   2. No
   3. I am not familiar with digital divide consults.

**Questions for all CAT therapists**

1. On a scale of 1-10, 1 being not confident at all and 10 being very confident, how confident are you with your use of technology?
2. What proportion of your patient load from 2019 have you been able to see either virtually or in person in 2020?

a. About the same as 2019

b. More than 2019

c. Less than 2019

1. Do you think there is a need for virtual delivery of CAT? Why or Why not?
2. Today, do you believe you have the proper infrastructure in terms of equipment to successfully deliver virtual CAT?
   1. Not at all
   2. Somewhat, but could be improved
   3. Yes
3. Today, do you have the administrative support to successfully deliver virtual CAT?
   1. Yes
   2. No
   3. Some support, but could use more
4. Today do you need additional training to deliver CAT virtually?
   1. Yes
   2. No
5. (If so) Do you have access to that training?
   1. Yes
   2. No
6. What types of further training or support related to virtual delivery would be helpful to you?
   1. Basic training (i.e. through TMS) on how to provide a virtual session
   2. Hand-on training
   3. Examples from other CAT therapists
   4. Other: ________________________
7. What types of further training or support related to virtual delivery would you suggest for VA CAT therapists in general?
   1. Basic training (i.e. through TMS) on how to provide a virtual session
   2. Hand-on training
   3. Examples from other CAT therapists
   4. Other: ________________________
8. Are there other issues related to potential delivery of virtual CAT that I have not asked about, which you think are relevant to note?
9. How has the COVID-19 outbreak affected you in the past month (choose all that apply)?
   1. Worked remotely or from home more than usual
   2. Worked more hours than usual
   3. Worked reduced hours
   4. Was not able to work
   5. Had difficulty arranging for childcare
   6. Incurred increased costs for childcare expenses
   7. Income or pay has been reduced
   8. Not paid at all
   9. Had serious financial problems

**Additional Questions for CAT therapists who have not used virtual delivery this year**

1. What clinical patient populations do you currently work with (choose all that apply)
   1. Mental health
   2. PTSD
   3. TBI
   4. Pain
   5. Hospice
   6. General Medical/ procedural support
   7. Geriatrics
   8. Other (specify) _________________
2. Are the majority of the interventions you deliver:
   1. Active interventions (where the participant is actively creating, such as drawing, making music, dancing)
   2. Receptive interventions (e.g. listening to music, viewing images)
   3. About equal active and receptive
3. Has your facility considered delivering CAT virtually?
   1. Yes
   2. No
4. Are you open to delivering CAT virtually?
   1. Yes
   2. No
